# Unbalanced Neonatal CD4^+^ T-Cell Immunity

**DOI:** 10.3389/fimmu.2014.00393

**Published:** 2014-08-27

**Authors:** Isabelle Debock, Véronique Flamand

**Affiliations:** ^1^Institut d’Immunologie Médicale, Université Libre de Bruxelles, Gosselies, Belgium

**Keywords:** vaccine, T helper subsets, Th2-biased response, defective Th1 response, follicular helper T-cells, Th17, Tregs

## Abstract

In comparison to adults, newborns display a heightened susceptibility to pathogens and a propensity to develop allergic diseases. Particular properties of the neonatal immune system can account for this sensitivity. Indeed, a defect in developing protective Th1-type responses and a skewing toward Th2 immunity characterize today the neonatal T-cell immunity. Recently, new findings concerning Th17, regulatory helper T-cell, and follicular helper T-cell subsets in newborns have emerged. In some circumstances, development of effector inflammatory Th17-type responses can be induced in neonates, while differentiation in regulatory T-cells appears to be a default program of neonatal CD4^+^ T-cells. Poor antibody production, affinity maturation, and germinal center reaction in vaccinated neonates are correlated with a limiting expansion of T_FH_ lymphocytes. We review herein the factors accounting for and the implications of the unbalanced neonatal helper T-cell immunity.

Pioneer experiments by Medawar, Billingham, and Brent showed that mice exposed to allogeneic cells at birth did not reject a graft expressing the same alloantigens (1). This transplantation tolerance or neonatal tolerance was associated with intrathymic clonal deletion of donor-specific cytotoxic CD8^+^ T-cells (2, 3). Further studies actually demonstrated that allospecific CD4^+^ T-cells differentiated into IL-4-producing Th2 cells in mice tolerating the allogeneic cells and tissues, while IL-2 and IFN-γ production by Th1 cells was defective (4–6). These discoveries indicated that neonates were not immunodeficient as first thought, since they were able to mount effective, while biased, immune responses. The neonatal stage was therefore considered as a transitory period characterized by an absence of memory cells and a slow adaptive immune response with tolerance features. Furthermore, the immune deviation to the Th2 subset and the defect in developing protective Th1-type responses could account for the higher susceptibility to infectious agents presented by neonates, as well as their propensity to develop allergic diseases in developed countries (7). This particular immunological status also led to consider the neonatal period as a beneficial time window to develop immune tolerance to specific antigens, like autoantigens.

The precise mechanisms underlying the polarization of the neonatal CD4^+^ T-cell compartment were delineated quite recently. When appropriately stimulated, neonates may achieve an immune response comparable to adult counterparts. Several works in mice first established that Th1-type responses could be induced in neonates with Th1-promoting inflammatory treatments, and brought some clues to explain the unbalanced Th1/Th2 neonatal immunity. This induction of Th1-cell development and, consequently, the modulation of the Th2-type response could be achieved by administration of IFN-γ itself (8), antigen-presenting cells (APCs)-derived IL-12 (9), dendritic cells (DCs) (10), live virus (11, 12), DNA vaccines (13), CpG nucleotides (14), or by CD40 triggering (15). These studies all suggested that particular properties of neonatal CD4^+^ T-cells and APCs could be involved in the skewed neonatal adaptive immune system.

Zaghouani and colleagues actually identified a mechanism contributing to the Th2 immune deviation in neonates at the molecular level (16). They demonstrated that mouse neonates could mount antigen-specific Th1 and Th2-type primary responses following antigenic stimulation. However, upon recall with antigen, neonatally stimulated Th1 cells died by apoptosis, while neonatally stimulated Th2 cells were not affected and mounted a secondary response. The apoptosis of primary Th1 cells was driven by the Th2 prototypic cytokine IL-4. To mediate this effect, IL-4 signaled through its alternative heteroreceptor composed of the IL-4Rα and IL-13Rα1 chains that is only expressed on neonatal IFN-γ-producing Th1 cells when IL-12p70-producing DCs are not present, thus favoring the development of neonatal Th2 cells. In line with this, studies have demonstrated the importance of age at the time of initial respiratory syncytial virus (RSV) infection in determining the pathophysiological responses. In human infants, a Th2-skewed immune response is particularly dominant in the lung in response to RSV and leads to severe bronchiolitis and subsequent asthma development with persisting respiratory dysfunction into adulthood. A significant up-regulation of IL-4Rα expression was recently detected on human cord blood CD4^+^ T-cells, in contrast to adult peripheral blood CD4^+^ T-cells, following *in vitro* RSV stimulation (17). Furthermore, the IL-4Rα-mediated reinforced Th2 response was confirmed *in vivo* upon neonatal infection in mice. Indeed, an increased expression of IL-4Rα on CD4^+^ T-cells (more pronounced on Th2 than on Th1 cells) from mice neonatally infected with RSV and reinfected with RSV as adults was observed. Thus, the neonatal RSV pathogenesis was associated with the expression of IL-4Rα on CD4^+^ T-cells that become functional upon RSV reinfection and positively control the Th2 response, the airway hyper-activity, and the inflammation. Although, here, the mechanism explaining the skewed Th2-dominant response is a selective higher induction of Th2 proliferation as a selective Th1 apoptosis triggered through the IL-4Rα/IL-13Rα1 heteroreceptor during reinfection in neonatally infected mice was not monitored (17).

Moreover, neonatal CD4^+^ T-cells display intrinsic epigenetic conformation within the Th2 genomic locus promoting the Th2 cytokines genes expression and the subsequent development of Th2 cells (18). In mice, the enhancer and regulatory region conserved non-coding sequence-1 (CNS-1) of the Th2 locus is indeed hypomethylated in thymic and peripheral neonatal CD4^+^ T-cells, allowing a rapid and high production of IL-4 and IL-13. In addition, as in adults, the transcription factors regulating Th1 and Th2 differentiations, T-bet, and STAT6, respectively, are already functional in early life (19). The neonatal helper T-cell compartment thus presents unique properties contributing to the unbalanced Th1/Th2 response in early life. This intrinsic bias was recently confirmed in human neonatal CD4^+^ T-cells. Indeed, a novel subset of naive CD31^+^CD4^+^ T-cells that express and store intracellularly an unglycosylated isoform of IL-4 was identified in infant adenoids (20). These IL-4 isoform-expressing cells are not present in adults and are suspected to be early-age restricted Th2 precursors’ cells that would spontaneously differentiate in mature IL-4 secreting Th2 cells. At the level of APCs, in mice, the absence of IL-12p70 (composed of IL-12p35 and IL-12p40 chains) producing neonatal DCs in the first days of life favors the expression of the alternative receptor of IL-4 on neonatal Th1 cells, rending them sensitive to IL-4-induced apoptosis, and promotes Th2-cell development (21). By day 6 after birth, a particular subset of CD11c^+^ CD8α^+^ IL-12-producing DCs that would allow a shift to Th1 immunity arises in newborn mice (21). These data confirmed the previously reported adult level of IL-12p70 secretion by 7-day-old splenic CD11c^+^ sorted DCs stimulated *in vitro* with TLR9 ligand (22). A defect in IL-12p70 secretion by neonatal monocyte derived DCs is observed in humans, a repressive chromatin state hampering IL-12p35 gene transcription (23). Furthermore, the promoter of the IFN-γ gene is hypermethylated in cord blood CD4^+^ T-cells, while neonatal CD8^+^ T-cells do not present this epigenetic modification. The *Ifng* promoter hypermethylation is associated with impaired production of this Th1 cytokine (24). Conversely, the IL-13 locus is maintained in an accessible chromatin architecture with the appearance of DNase I hypersensitivity sites and extensive DNA demethylation (25) that could account for the elevated IL-13 expression by human neonatal CD4^+^ T-cells upon TCR triggering (26). An unbalanced Th1/Th2 adaptive immunity might therefore be also at play in human neonates.

## Neonatal Th17 Adaptive Immunity

With this demonstration of the Th2-skewed immunity and the possible induction of Th1 immunity under appropriate conditions in newborns, the question about the capacity of neonates to develop other helper T-cell responses has been raised. In particular, some attention has been paid to the development of inflammatory Th17-type responses that could compensate for the defective neonatal Th1 immunity. Th17 cells were initially identified in mouse models of autoimmunity (27, 28) and established as an independent CD4^+^ helper T-cell subset soon after their identification (29, 30). Murine studies elucidate their differentiation process as initiated in the presence of two cytokines, TGF-β and IL-6 (31–33) or TGF-β and IL-21 (34–36), although the need of TGF-β has been challenged (37). TGF-β produced by murine Th17 cells is also necessary for stabilizing in an autocrine manner the commitment of the Th17 cell lineage (38). The Th17 cell development is sustained in the presence of IL-23, a member of the IL-12 family composed of the IL-23p19 chain and the IL-12p40 chain that is shared with IL-12. Th17 cells typically produce IL-17A, IL-17F, and IL-22, which can recruit neutrophils and confer host protection against extracellular bacteria and fungi at epithelial barriers (39). Study of the Th17 cell development in neonates is of particular interest as preterm infants are at higher risk of developing bacterial and fungal infections. As for every helper T-cell subset (40), the Th17 cell development is negatively regulated by the key cytokines of the other subsets. Indeed, the presence of IFN-γ or IL-4 in the naive CD4^+^ T-cells environment inhibits their differentiation in Th17 lymphocytes (41).

In the context of the neonatal Th2-biased immune response, the inhibitory effect of IL-4 on the development of inflammatory Th17-type responses could represent a major regulation mechanism. A first study on experimental autoimmune encephalomyelitis showed that neonates could mount mixed Th1 and Th17-type autoantigen-specific immune responses (42). However, there were fewer IL-17-producing cells in neonates compared to adult mice, which is in agreement with another report (43). The question of the direct inhibition of neonatal Th17-type responses by IL-4 was addressed in the neonatal tolerance context (44). Mouse neonates immunized with allogeneic cells were also submitted to IL-4 deprivation that inhibited the development of the allospecific Th2-type response (45) and allowed the mRNA up-regulation of Th17-type cytokines and the master regulator of the Th17 pathway RORγt (46), thereby favoring the development of alloreactive IL-17A-producing Th17 cells (44). Likewise, neutralization of IL-25, a Th2-prone cytokine, following murine neonatal infection with pneumonia virus, which belongs to the same family as RSV, reduces some key features of asthma and promotes a Th17-type response. In the infected lung, IL-25 is expressed locally by the airway epithelial cells and can initiate the Th2 differentiation in an IL-4-dependent manner (47). Recently, other works in mice suggested that neonates could mount IL-17A-associated immunity upon infection with the enteropathogen *Yersinia enterolitica* (48) or anti-mycobacterial vaccination with particular adjuvant (49, 50). In humans, stimulation of cord blood naive T-cells, particularly CD8^+^ T-cells, with IL-23 induces the expression of IL-23 receptor and the production of IFN-γ and IL-17 (51). In addition, a strong production of IL-23 by neonatal DCs, as well as monocytes, has been reported (52, 53), suggesting that there is no defect in the expression of the IL-12p40 chain, and that human neonates could develop effective Th17 immunity. Accordingly, IL-17-producing cells originated exclusively from cord blood CD161^+^CD4^+^ T-cell progenitors were detected in response to IL-1β and IL-23 (54). In agreement with this notion, an *in vitro* study suggested that naive CD4^+^ T-cells from preterm and term infants exhibited higher expression of IL-23R, RORC, and STAT3 mRNA than adult counterparts and were accordingly capable of differentiating in Th17 cells under polarizing conditions (with added IL-1, IL-6, IL-23, and TGF-β) and stimulation with anti-CD3 and anti-CD28 antibodies (55). However, in another culture system with anti-CD3 antibodies and cord blood monocytes as viable APCs, naive neonatal CD4^+^ T-cells were defective in Th17 development and addition of IL-1β, IL-6, and IL-23 only partially restored the IL-17 production. This defect was correlated with a clear reduced RORC2 mRNA levels in neonatal CD4^+^ T-cells. Human neonatal T-cells could therefore be considered as having intrinsic mechanism that prevents Th17 differentiation through the regulation of RORγT expression (56). Interestingly, a recently described cord blood population of CD4^+^ T-cell precursors with an effector memory phenotype was shown to display features of IFN-γ producing Th1-like cells, IL-4 producing Th2-like cells but IL-17-deficient Th17-like cells unless they were stimulated with IL-1β and IL-23 (57).

We may therefore consider that under strong TCR/CD28 and cytokine polarizing signaling with no dominance of Th2-type immunity, development of effective Th17 cells can be induced in neonates.

## Neonatal Regulatory T-Cells and Maternal Microchimerism

Since the Th2 bias impairs Th1 and Th17-type immune responses in early life, neonatal immunity is typically considered to exhibit an anti-inflammatory profile. In this context, one may suspect that regulatory T-cells (Tregs) play an important role in early life to maintain this anti-inflammatory status. Indeed, it was shown that the lack of cytotoxic activity by CD8^+^ T-cells reactive to allogeneic cells is controlled by CD4^+^ CD25^+^ Tregs (58). Additionally, Tregs and Th17 cells have a reciprocal development pathway. In the presence of TGF-β, their transcriptional master regulators, i.e., Foxp3 for Tregs and RORγT for Th17 cells, respectively, are upregulated. In the absence of proinflammatory cytokines like IL-6 (in mice), or IL-6, IL-1β, and IL-23 (in humans), Foxp3 dominates RORγT function and prevents Th17 development. Such mechanism could impair the differentiation of Th17 cells in early life.

More recently, mechanism about the contribution of Tregs in neonatal immunity was proposed. Murine neonatal CD4^+^ thymocytes and T-cells actually seem to be prone to intrinsically differentiate into suppressive Foxp3^+^ Treg cells (59), even though newborn mice display low frequency of Tregs (60). Strikingly, TCR triggering alone was shown to be sufficient to induce enhanced Tregs differentiation in neonates. Exogenous addition of IL-2 or TGF-β, both required to differentiate adult Tregs, was indeed unnecessary (59). In humans, activation of naive CD45RO^−^ CD25^−^ CD4^+^ T-cells in the presence of APCs showed increased numbers of functional Foxp3^+^ cells presenting an enhanced expression of PD-1 molecules in cord blood than in adult peripheral blood (61). These studies suggest that Tregs are functional in early life. While neonatal Tregs are thought to be essential for the early life induction of immune tolerance to self antigens, their presence can conversely impair neonatal effector and protective responses to vaccines and pathogens, as proposed by a report on neonatal HSV infection in mice (62) and during *Plasmodium falciparum* infection in humans. Indeed, CD4^+^ CD25^+^ Foxp3^+^ T-cells downregulated IFN-γ production of cord blood monocytes in response to the parasite through production of IL-10 (63).

Furthermore, development of Tregs seems to occur very early in life. During pregnancy and weaning, the developing immune system actually faces allogeneic stimulation. The fetus and neonate indeed encounter maternal allogeneic cells that present non-inherited maternal alloantigens (NIMA) and are transferred across the placenta and through ingestion of breast milk (64, 65), leading to the establishment of maternal microchimerism. This exposure to maternal alloantigens is associated with the induction of Foxp3^+^ Tregs specific of NIMA in newborn mice (66, 67). Development of tolerogenic Tregs upon NIMA stimulation is also described in human fetuses (68). However, exposure to NIMA can also lead to priming of cytotoxic and Th1 T-cell responses when the dose of NIMA encountered is low (69), high doses being associated with the development of Tregs (66, 70). Maternal microchimerism thus influences adaptive immunity in early life. Furthermore, it was proposed in a model of juvenile diabetes that maternal microchimeric cells could initiate inflammation to autoantigens and shape the fetal immune response (71). Besides this, the allogeneic stimulation of fetal and neonatal immune system by NIMA-expressing cells is likely to be the first immunological event of life.

## Neonatal Follicular T Helper Cells

Another important question in neonatal T-cell immunity concerns the status of follicular helper T-cells (T_FH_). This CD4^+^ helper T-cell subset was first described in human tonsils (72, 73) and is defined as the specialized provider of help to B-cells (74). Indeed, T_FH_ cells play a key role in germinal center (GC) reaction, isotype class switching and antibody affinity maturation through their interactions with cognate B-cells. The development of effective T_FH_ cells requests the presence of various molecules, including Bcl6, CXCR5, PD-1, IL-6, and IL-21 (74). Among these markers, Bcl6 is the transcriptional master regulator of T_FH_ cells essential for the expression of the T_FH_ cell genetic program (75–77). Notably, Bcl6 induces the expression of the chemokine receptor CXCR5 (78), which is crucial for the positioning of T_FH_ cells in B-cell follicles and GCs in secondary lymphoid organs. T_FH_ cells produce IL-21, a cytokine acting on B-cells and on T_FH_ cells themselves via an autocrine loop (79–81). With regards to the relationship between T_FH_ cells and other CD4^+^ T-cell subsets, a negative regulation exists as Bcl6 represses the expression or function of the master regulators of other Th-cell lineages, i.e., T-bet (Th1), GATA3 (Th2), and RORγt (Th17) (75–77). However, this effect appears to be partial, as T_FH_ cells can produce IFN-γ (82), IL-4 (83, 84), or IL-17 (85), hallmarks of Th1, Th2, and Th17 cells.

Two recent studies in mice sought to determine if newborns can mount effective T_FH_ cell responses (86, 87). Both works showed that CD4^+^ CXCR5^+^ PD-1^+^ T_FH_ cells can be induced in mice neonates subjected to antigenic immunization, but that their full development is impaired. Indeed, neonatal T_FH_ cells expressed lower levels of Bcl6 and IL-21 and were poorly located in GCs, while they were highly present in interfollicular regions (86). This localization outside of GCs correlates with the limited expansion and differentiation in GC T_FH_ cells of newborn T_FH_ cells (87). Corresponding to this limited T_FH_ cell response, the humoral immune response of immunized neonates was defective, as revealed by a reduced antibody production and maturation and a decreased and delayed formation of GCs (86).

Both studies also indicated IL-4 expression by neonatal T_FH_ cells. The role of IL-4 in the emergence of T_FH_ cells in newborns was addressed by Debock et al. (86). It was shown that this cytokine promotes the development of T_FH_ cells in early life. Indeed, the proportion of T_FH_ cells in immunized IL-4-deficient newborns was greatly reduced. Moreover, in the absence of IL-4, neonatal T_FH_ cells highly expressed RORγt and IL-17, features of Th17 cells. This particular phenotype was associated with an enhanced production of IgG2a in immunized neonates, suggesting that IL-4 impacts the genetic profile and the B-cell help specificity of neonatal T_FH_ cells.

The finding that T_FH_ cells are present and functional in early life, although in a limited extent compared to adults, is of interest to improve neonatal and pediatric vaccines. Indeed, in the context of defective follicular DCs and B-cells, the other partners of humoral immunity, in newborns (88), early priming of neonatal T_FH_ cells could promote the induction of high-affinity antibody responses when the other cellular actors become functional. Interestingly, administration of a CpG-adjuvanted vaccine in mouse neonates circumvented the limited expansion and development in GC T_FH_ cells of neonatal T_FH_ cells (87), suggesting that this could represent a beneficial strategy for efficient vaccination of newborns.

## Concluding Remarks and Future Prospects

**Figure 1:**
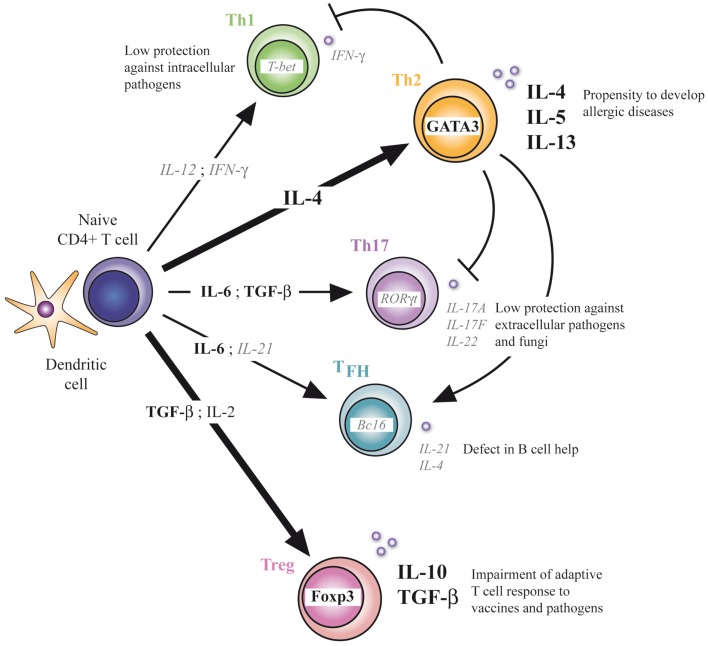
**CD4^+^ T-cell differentiation in early life**. Defect in Th1, Th17, and TFH differentiation and enhanced Th2 and Treg differentiation characterize the neonatal T-cell immunity. At the molecular level, respective transcriptional factors and cytokines are positively (bold) or negatively (italic) regulated. Implications of this unbalanced adaptive immunity are depicted.

Our understanding of neonatal helper T-cell immunity was greatly improved in the last years. Precise mechanisms underlying the profound defect of Th1 immunity and the subsequent Th2-biased polarization in early life were indeed identified at the molecular and cellular levels. This imbalance in neonatal T-cell responses may imprint development of adult T-cell responses as illustrated in a study showing the higher susceptibility to allergic inflammation of mice neonatally immunized with a pro-Th2 vaccine and a protection when pro-Th1 vaccine was used (10). In addition to this, neonates also appear capable of mounting inflammatory Th17-type responses when properly stimulated. This could be a potential target to ensure neonatal protection to pathogens and to prevent development of allergic diseases, as neonatal Th17 immunity could compensate for the lack of Th1-type immune responses and oppose the Th2 pathway. However, caution should be taken as induction of strong Th17-type responses could possibly break the induction of immune tolerance to self antigens and favor the development of autoimmunity. The default generation of Tregs in neonates could furthermore promote the Th17 pathway by producing one of its differentiation cytokine, TGF-β (31–33), which could lead to an uncontrolled inflammatory response. Notwithstanding this, Treg cells appear to play a critical role in the developing immune system, contributing to the neonatal general anti-inflammatory status and limiting protective immune responses developed by newborns. The presence and functionality of T_FH_ cells in neonates were also recently delineated. These providers of B-cell help are induced upon vaccination in early life, but their development is limited compared to adults. This correlates well with the defective humoral immune responses observed in newborns. Targeting this particular T-cell population with appropriate adjuvants could enhance vaccine responses in newborns and therefore reduce the need of several booster doses in early life to reach effective IgG responses while increasing their resistance to vaccine-preventable life-threatening infections like tuberculosis, malaria, and HIV (Figure 1).

Regarding the aspects that still need to be addressed in neonatal T-cell immunity, the phenotype and the differentiation pathway of neonatal Th17 cells require further study. TGF-β-stimulated Th17 cells can indeed produce IL-17 and IL-10, thus displaying a more regulatory phenotype, while Th17 cells differentiated in the presence of IL-23 seem to be more pathogenic, these inflammatory properties being due in part to their production of IFN-γ (37). Since IL-23 is highly produced in human neonates, the inflammatory Th17 cell subpopulation could be more represented in newborns. However, considering the neonatal regulatory context, regulatory Th17 cells producing IL-10 and limiting inflammatory response could arise preferentially. In line with this hypothesis, neonatal B-cells secreting IL-10 have been shown to control DCs response upon TLR triggering (89, 90).

More clarification is also needed for the development and function of T_FH_ cells in early life, notably with regards to their potential link with the other Th-cell subsets. While there are now some insights in the relationship between the newborn T_FH_ cells and the Th2 and Th17 populations (86), the potential link of neonatal T_FH_ cells with Tregs is also of interest since follicular Tregs expressing CXCR5, PD-1, and CTLA-4 or GITR have been described (91). Also, the role in early life of recently described T_FH_ cell regulators like the transcription factor achaete-scute homolog 2 (92) or the Notch signaling (93), as well as of molecules involved in the T_FH_ cell/B-cell interactions such as the SAP/SLAM family of molecules (74) or BLyS (94), requests further attention. In addition, better phenotypical characterization of neonatal T_FH_ cells could lead to the identification of potential new targets for vaccination.

Another intriguing question is the potential development of IL-9-producing Th9 cells in neonates, since they are induced in the presence of IL-4 and TGF-β (95). Considering the heightened production of IL-4 and the default generation of Tregs displayed by neonates, Th9 cells could represent an important Th-cell subset in newborns.

Finally, epigenetic modifications hampering the Th1 pathway and favoring the Th2 response have been described in neonates. It would be interesting to determine, which transcription factors and histones and DNA modifying enzymes are responsible for the *Il12p35* and *Ifng* genes repressive chromatin state, and if a particular chromatin conformation is also present in the *Il17a*, *Il10*, and *Foxp3* genes. This could provide therapeutic targets to induce protective immunity in early life.

## Conflict of Interest Statement

The authors declare that the research was conducted in the absence of any commercial or financial relationships that could be construed as a potential conflict of interest.
